# Prognosis of patients with suspected pulmonary embolism in Buenos Aires: a prospective cohort study

**DOI:** 10.1186/1471-2466-14-200

**Published:** 2014-12-15

**Authors:** Fernando Javier Vazquez, María Lourdes Posadas-Martínez, Fernán González Bernaldo de Quirós, Diego Hernan Giunta

**Affiliations:** Department of Internal Medicine, Hospital Italiano de Buenos Aires, Buenos Aires, Argentina; Research Area in Internal Medicine, Hospital Italiano de Buenos Aires, Buenos Aires, Argentina

**Keywords:** Pulmonary embolism, Prognosis, Outcome, Suspected pulmonary embolism, Mortality, Comorbidities

## Abstract

**Background:**

The prognosis of patients with suspected pulmonary embolism (PE) in whom PE has been ruled out (RPE) is unclear. We aimed to evaluate survival and diagnosis of new cancer in suspected PE patients at follow up.

**Methods:**

A prospective cohort study nested in a prospective Institutional Registry of Venous Thromboembolic Disease was performed between 2006 and 2011. This study was designed to evaluate all consecutive, incident cases of suspected PE in adults. The study was performed at the Hospital Italiano de Buenos Aires, a tertiary level hospital, in hospitalized patients and outpatients. Suspected PE cases were collected using a computerized system that alerts whenever a physician requests pulmonary angiography, angiotomography, or ventilation-perfusion scintigraphy. PE was defined by pre-specified criteria and RPE was defined when diagnostic tests were negative for PE.

**Results:**

We included 1736 cases of suspected PE. The prevalence of PE was 29% (n = 504). There was no difference in the overall survival at 30 days and follow-up between PE and RPE patients. The presence of provoked or unprovoked venous thromboembolic disease in these patients did not affect survival. The main causes of death were PE in the confirmed PE group (60%), and neoplasm (42%) and sepsis (37%) in the RPE group. Survival at 90 days was 63% for PE (95% CI 58–67%) and 67% for RPE patients (95% CT 64–69%). At follow-up, there was no difference in diagnosis of new cancer between PE and RPE patients (2% vs 2%, p = 0.82), even when taking into account the unprovoked group.

**Conclusions:**

Even when the main cause of death in PE patients is PE itself, the overall mortality is similar between PE and RPE patients. The reason for this finding could be because of the more frequent and severe comorbidities in RPE than in PE patients.

**Trial registration:**

HomeClinicalTrial.gov: NCT01372514

## Background

Pulmonary embolism (PE) causes 5–10% of inpatient deaths, representing the main cause of preventable deaths in hospitalized patients [1]. Only 15–30% of suspected PE cases are finally confirmed [2–12]. There are few reports comparing the short-term and long-term mortality in patients with suspected PE, particularly for those in whom PE has been ruled out (RPE) [13–18]. PE has a short-term (30 days) and long-term mortality, which varies between 5% and 30% [3, 17, 19–24]. Clinical manifestations of PE are often nonspecific, and therefore, PE is challenging to diagnose. In fact, the risk factors for venous thrombosis disease (VTE) and comorbidities in confirmed PE and RPE patients are similar [13], and may be determinant in the prognosis of patients with suspected PE [13].

While the mortality of confirmed PE is not high when the disease is promptly recognized and treated, PE may have high mortality when it is massive or is not suspected in the early stage. The prognostic information in patients with confirmed PE versus those with RPE is limited. We believe that all patients with suspected PE have a similar prognosis because of the burden of comorbidity, independent of the diagnosis of PE. This may have implications for providing information on the fragility of this group of patients.

We performed a prospective cohort study to evaluate the overall mortality in patients with suspected PE at a tertiary level hospital. We compared short-term (30 days) and long-term (2 years) mortality between confirmed PE and RPE patients. Secondary objectives were to evaluate the recurrence of VTE in confirmed PE, new VTE in RPE, bleeding, and diagnosis of new cancer during follow-up. Subanalysis was performed considering three groups according to the presence or absence of risk factors for VTE (cancer, and provoked and unprovoked VTE), which were present in each patient at the time of the suspected PE.

## Methods

We performed a prospective cohort study nested in the prospective Institutional Registry of Venous Thromboembolic Disease (IRTD Home ClinicalTrial.gov NCT01372514) between June 2006 and May 2011. This study was designed to evaluate all consecutive incident cases of suspected PE at the Hospital Italiano de Buenos Aires [25]. This hospital is a tertiary level hospital with 675 beds, 42,700 admissions, and almost 3 million ambulatory consultations each year.

### Population

Suspected PE was considered if any patient (in the hospitalized or ambulatory setting) was assessed for PE with a diagnostic test (computed tomography pulmonary angiography [CTPA], ventilation-perfusion scintigraphy, or pulmonary angiography).

The IRTD inclusion criteria were as follows: (1) all consecutive inpatients and outpatients older than 16 years of age; (2) patients who were evaluated with a diagnostic test (CTPA, ventilation-perfusion scintigraphy, or pulmonary angiography) by the attending physician to confirm or rule out PE because of the presence of signs and/or symptoms suggestive of PE (defined as acute onset of new or worsening shortness of breath, tachycardia, or chest pain without any other obvious etiology); and (3) confirmed deep vein thrombosis (DVT) with an objective diagnostic test. Patients were excluded from the IRTD for the following reasons: (1) patients were tested for any other reason than suspected VTE; (2) patients were unable to provide informed consent or refused to participate; and (3) patients who only visited our hospital for a diagnostic study, but were treated at other centers. For this study, we included only those patients with suspicion of PE who were already included in the IRTD.

Possible cases were collected for the IRTD using a computerized alert that was generated whenever a physician requested an imaging study for VTE among adult patients. Data collection was carried out prospectively by medical students who were specifically trained for this study. They interviewed the patient and the treating physician using a standardized data collection form. Baseline evaluation of all patients, including the Wells score [26, 27], was performed during the first 24 hours since suspicion of PE, without knowing the diagnostic test results.

PE was confirmed if CTPA or angiography showed filling defects in a sub-segmental or larger pulmonary artery, and was present in at least two consecutive images, or there was a high or intermediate probability of PE in ventilation-perfusion scintigraphy, associated with a likely Wells score probability [25, 27]. All others cases were considered as RPE [27] and an alternative final diagnosis was always confirmed.

In this study, patients were categorized according to risk factors for VTE at the time of suspected PE for the following: 1) active cancer (i.e., patients who were diagnosed with cancer or who had received cancer treatment in the last 6 months); 2) provoked, which was defined as patients with any risk factor for VTE (transient or not, except for cancer) detected at the time of suspected PE [28], including reduced mobility, advanced age (70 years old or older), acute medical illness, recent major surgery or trauma, spinal cord injury, obesity, hormone replacement therapy, oral contraceptives, a cast or immobilizer, pregnancy or postpartum period, recent travel (more than 6 hours during the last 30 days), and thrombophilia; and 3) unprovoked, which was defined as patients in whom no evident risk factor for VTE was detected at the time of suspected PE [29–31].

Follow-up was performed through medical electronic records and structured phone call interviews, to assess overall mortality and complications. All of the study patients were contacted every 6 months following the suspected PE episode. The cause of death was determined systematically by the treating physician and the principal investigator.

Complications of VTE were considered as follows: 1) major bleeding, which was defined by the International Society of Thrombosis and Hemostasia [32]; 2) a new VTE event (i.e., new symptomatic DVT and/or PE event that was objectively confirmed; and 3) diagnosis of new cancer (i.e., diagnosis of neoplasm 30 days after the suspected PE event during follow-up).

This study was observational, and all diagnostic and therapeutic medical decisions reflected current medical practice. This study was approved by the ethics committee of the Hospital Italiano de Buenos Aires (protocol # 995). Informed consent was obtained from all participants.

### Statistical analysis

Descriptive analysis for continuous variables is shown as mean and standard deviation or median and interquartile range, according to the observed distribution. Categorical variables are expressed as absolute number and percentage. Comparisons between groups at baseline were performed with the chi-square test for categorical variables and the Mann–Whitney U test for continuous variables.

For patients with SPE, survival was calculated with the Kaplan–Meier estimator. Median survival time was expressed with its 95% confidence interval (95% CI). Survival distribution according to VTE risk factor categories (cancer, provoked, and unprovoked) was also estimated with the Kaplan–Meier estimator. Survival curves were compared between the PE and RPE groups with the Mantel–Cox test.

Significance was defined as p < 0.05. All data analyses were performed with IBM SPSS software, version 19 (SPSS, Chicago, IL).

## Results

### Prevalence of PE

**Figure 1:**
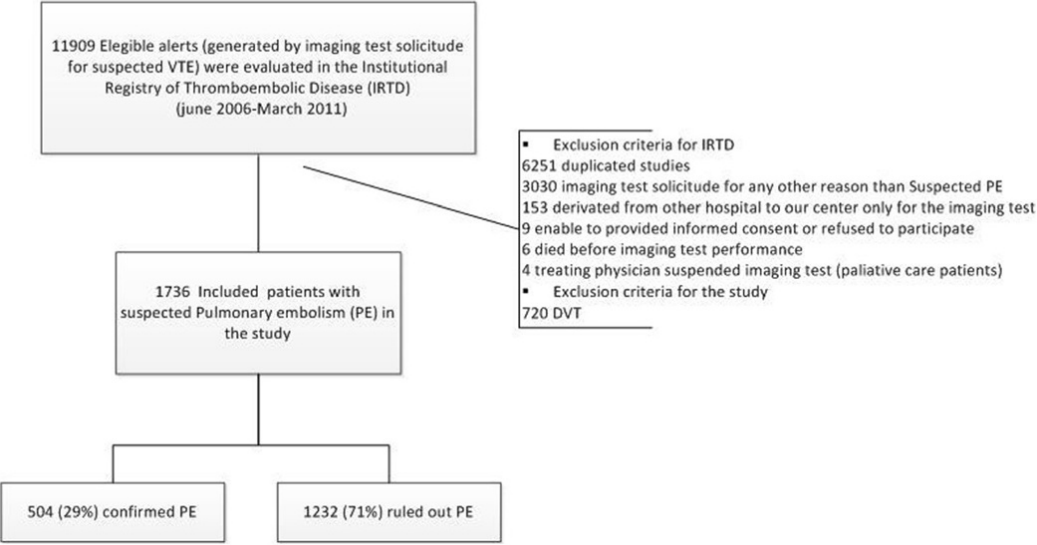
**Flow diagram of the study.**

A total of 1736 patients were included for suspected PE in the IRTD, and the prevalence of PE was 29% (n = 504) (Figure 1). The prevalence of PE according to the main risk factor was 29% (201/688) in the active cancer group, 29% (253/854) in the provoked group, and 26% (50/194) in the unprovoked group.

### Alternative etiological diagnoses in RPE

The most common etiological diagnoses for RPE patients were intrathoracic neoplasm (23%, 288/1232), congestive heart failure (CHF) (21%, 256/1232), pneumonia (17%, 206/1232), chronic obstructive pulmonary disease (COPD) (9%, 118/1232), sepsis (7%, 86/1232), acute coronary syndromes, dissection or aneurysm of the aorta (3.5%, 43/1232), and others (6%, 74/1232).

### Main characteristics of the patients

Patients with confirmed PE had a higher body mass index, and a higher proportion of nephrotic syndrome and a history of VTE or major surgery within the last 30 days. RPE patients had an increased frequency of CHF, coronary heart disease, and heparin prophylaxis prior to the suspected PE event (Tables 1 and 2). The simplified Wells score, which was performed a priori to the diagnostic tests, accurately discriminated between patients who finally had confirmed PE and RPE (median, 4.5 points [95% CI 3–7] and 2.5 points [95% CI 1–4], respectively, p < 0.01).

**Table 1:** **Baseline characteristics of the two groups**

	Confirmed PE	Ruled out PE	p
	n = 504	n = 1232	
Median age (IQR, interquartile range) years	71 (60–80)	71 (58–81)	0.36
Sex-Female, % (n)	58% (291)	60% (736)	0.45
Median stay (IQR) days	8 (5–15)	8 (4–14)	0.78
Comorbidities			
Diabetes	12% (61)	15% (187)	0.09
Hypertension	63% (313)	67% (816)	0.09
Smoking	13% (64)	12% (144)	0.56
Heart Failure	10% (50)	20% (241)*	0.001
Coronary heart disease	10% (47)	14% (166)*	0.016
Dyslipidemia	48% (242)	45% (548)	0.2
Cerebrovascular Accident	5% (25)	6% (75)	0.47
Active neoplasia	40% (201)	40% (487)	0.89
Liver disease	1% (6)	1% (1)	0.50
Nephrotic syndrome	2% (7)	1% (4)*	0.012
Charlson Score*	2 (0–3)	2 (1–4)*	0.025
Immobilization within 4 weeks	64% (304)	64% (732)	0.98
Median Body Mass Index (IQR) kg/m^2^	29 (25–31)	28 (24–30)*	0.05

*p < 0.05. Categorical variables were compared with the chi^2^ test and continuous variables with the Mann Whitney U test.

**Table 2:** **Risk factors by group**

	Confirmed PE	Ruled out PE	p
	n = 504% (n)	n = 1232% (n)	
Central venous catheter	10% (50)	10% (114)	0.69
Recent major surgery (<30 days)	30% (150)	23% (276)*	0.001
Active cancer disease	40% (201)	40% (487)	0.89
Prior VTE event	16% (78)	10% (121)*	0.014
Immobilization within 4 weeks	64% (304)	64% (732)	0.98
Pharmacological thromboprophylaxis^$^	14% (70)	20% (241)*	0.03
Recent trauma (<30 days)	8% (39)	6% (76)	0.24
Unprovoked	9.9% (50)	11.7% (144)	0.29
Medical reason for hospitalization	31% (55)	34% (165)	0.5
OC/HRT	2% (8)	2% (19)	0.94
Recent travel >6 hours	11% (54)	10% (116)	0.43

^$^The use of pharmacologic thromboprophylaxis was defined as the use of low molecular weight heparin, unfractioned heparin (UFH) or fondaparinux (2.5 mg once daily) before being evaluated for suspected PE. *p < 0.05. Categorical variables were compared with the Chi^2^ test and continuous variables with the Mann Whitney U test. *Abbreviations:* *OC* Oral contraceptives, *HRT* hormone replacement therapy.

### Survival of patients with PE and RPE during follow-up

**Figure 2:**
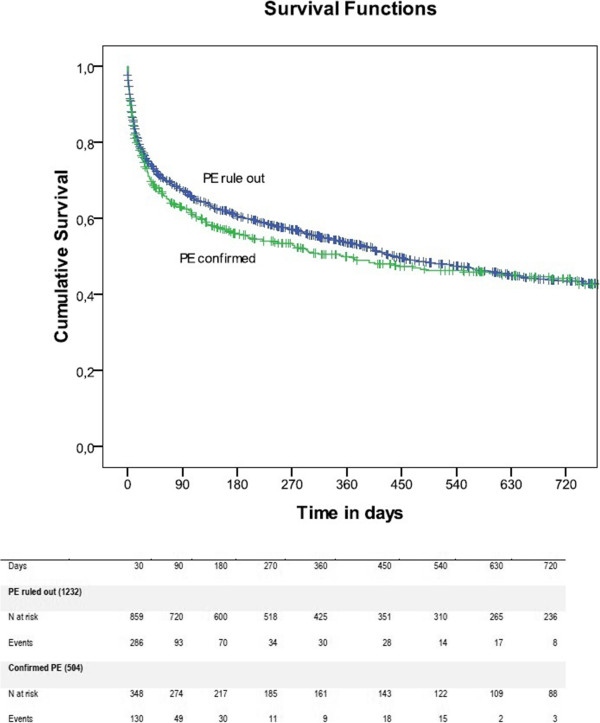
**Kaplan Meier survival curve of confirmed and ruled out PE during follow up.**

The cumulative proportion surviving at 30 days for RPE and confirmed PE was 0.76 (95% CI 0.73–0.78) vs 0.73 (95% CI 0.69–0.76), at 90 days it was 0.67 (95% CI 0.64–0.69) vs 0.63 (95% CI 0.58–0.67), at 360 days it was 0.53 (95% CI 0.50–0.55) vs 0.49 (95% CI 0.44–0.54), and at 720 days it was 0.43 (95% CI 0.40–0.47) vs 0.43 (95% CI 0.38–0.47). There was no significant difference in the distribution of survival between the groups (p = 0.52). Figure 2 shows the Kaplan–Meier curve for PE and RPE patients.

The overall median survival time was 421 days (95% CI 334–508). The median survival time was 438 days in RPE patients (95% CI 343–533) and 351 days in confirmed PE patients (95% CI 153–554). At 2 years of follow-up, 162 (9.3%) patients were lost to follow-up because of impossibility of telephone contact. There was no significant difference in the number of patients who were lost to follow-up between confirmed PE patients (52/504, 10%) and RPE patients (110/1232, 9%, p = 0.37, Table 3). Those who were lost to follow-up were younger and healthier (less likely to have cancer, immobilization and comorbidities) than those who had complete follow-up.

**Table 3:** **Baseline characteristics between complete and lost follow up patients**

	Complete follow up	Lost of follow up	p
	n = 1573	n = 162	
Median age, years	68	63	<0.001
Sex-Female, %	41%	39%	0.60
Median stay, days	8	8	0.67
Comorbidities			
Diabetes	13.9%	19.3 %	0.07
Hypertension	65.3%	66.3%	0.81
Smoking	11.9%	16.5%	0.09
Heart Failure	16.9%	15.4%	0.62
Coronary heart disease	12.5%	11.2%	0.64
Dyslipidemia	45.6%	48.8%	0.45
Cerebrovascular Accident	5.8%	5.6%	0.60
Active neoplasia	40.9%	24.1%	<0.01
Nephrotic syndrome	0.8%	2.9%	0.21
Charlson Score*	2	1	<0.001
Immobilization within 4 weeks	37%	24.8%	0.04
Median Body Mass Index, kg/m^2^	27	27	0.04

* p < 0.05. Categorical variables were compared with the chi^2^ test and continuous variables with the Mann Whitney U test.

There was no significant difference in the distribution of survival between PE and RPE patients, when adjusted for the presence of the main risk factor for VTE (cancer, provoked, and unprovoked, p = 0.73). Survival in cancer patients was worse than that in the other two groups. Table 4 shows survival in PE and RPE patients stratified by the main risk factor. The most frequent causes of death at 90 days in patients who died after a confirmed PE event were fatal PE (60%), sepsis (22%), and neoplasm (16%). The most frequent causes of death in RPE patients were neoplasm (42%) and sepsis (37%). Survival analysis based on the main risk factor category at the time of suspected PE (cancer, provoked, or unprovoked) failed to show any significant difference (Table 4).

**Table 4:** **Survival by category of risk factor**

Survival	Active cancer N = 688		Provoked N = 854		Unprovoked N = 194	
	Confirmed PE (201)	Ruled out PE (487)	Confirmed PE (253)	Ruled out PE (601)	Confirmed PE (50)	Ruled out PE (144)
	S (95% CI)	S (95% CI)	S (95% CI)	S (95% CI)	S (95% CI)	S (95% CI)
**1 Year**	0.32 (0.24-0.39)	0.32 (0.27-0.36)	0.61 (0.54-0.67)	0.69 (0.64-0.75)	0.57 (0.42-0.71)	0.59 (0.50-0.67)
**2 Years**	0.26 (0.18-0.33)	0.24 (0.19-0.28)	0.53 (0.45-0.60)	0.57 (0.53-0.60)	0.51 (0.34-0.67)	0.49 (0.49-0.59)

S, Survival; CI: confidence interval; p < 0.05.

### Complications at follow-up

PE patients had more recurrent VTE episodes compared with RPE patients (11% vs 5%, p < 0.0001). Major bleeding was significantly higher in PE patients compared with RPE patients (7% vs 2%, p < 0.0001). The incidence of newly diagnosed malignancies was similar in PE and RPE patients in the entire cohort (2% vs 2%, p = 0.82), and in the unprovoked subgroup in which only two new cancers were diagnosed in PE patients (p = 0.40).

## Discussion

This study showed that patients with suspected PE had high overall short-term and long-term mortality rates, regardless of confirming or ruling out the diagnosis of PE. Patients with confirmed PE in our study had similar mortality rates to those reported in the literature [19, 23, 33]. The similarity in mortality between PE patients (27%) and RPE patients (24%) at 30 days after suspected PE was most likely due to direct mortality of confirmed PE during the acute period and the high comorbidity of both groups, as shown in other studies [13].

### Mortality of patients with confirmed PE

The International Cooperative Pulmonary Embolism Registry (ICOPER) and Longitudinal Study of Thromboembolic Etiology (LITE) studies reported short-term mortality rates for confirmed PE, similar to our findings for confirmed PE. In the ICOPER study, the main cause of death was PE, and in the LITE study, mortality was higher in patients with cancer [34]. Heit et al. [35] reported a slightly higher mortality than our study (28%) after 30 days, which was likely explained by the inclusion of PE cases that were identified at autopsy, without clinical suspicion of PE before death. In our study, autopsies of patients with obvious causes of death were not included. Anderson et al. [36] reported a lower mortality than our study (12%) in confirmed PE, but they only included patients who died during hospitalization. Flinterman et al. [24] reported higher 5-year mortality rates in patients with VTE than in controls. However, this finding can be explained because the controls did not present with suspected PE, but were relatives of patients with VTE or were patients matched for age and sex. In addition, these controls were younger with a lower prevalence of cancer, CHF, and COPD compared with VTE patients. The study by Flinterman et al. [24] has two features that are similar to our study: the most frequent causes of death (cancer, CHF, and PE), and death, which was more frequent in cancer patients than in the other categories of risk factors.

We detected a non-significant trend of early death in patients with confirmed PE, which could be explained by acute hemodynamic failure at onset and early recurrence of fatal PE or fatal bleeding related to anticoagulant therapy. Long-term mortality after PE is probably affected by comorbidities. Another important finding in our study was that, regardless of the category of VTE risk factor, patients had a similar risk of death, irrespective of whether the diagnosis of PE was confirmed or ruled out. Additionally, this risk was maintained in the long term follow-up. This could be potentially explained by the high burden of comorbidities, mainly cancer and sepsis.

### Mortality of RPE patients

Unlike patients with confirmed PE, information on clinical features, evolution, and mortality in patients with RPE is scarce. van Beek et al. [37], Akram et al. [13], and Bertoletti et al. [38] reported a lower mortality rate of PE than in the current study, but the main causes of death were similar (neoplasm and CHF). The higher rate of mortality in our study could have been due to baseline characteristics (older age and comorbidities, particularly cancer) and the follow-up time [38].

Roy et al. [18] reported a similar rate of prevalence of PE to our study. For those patients who died, fatal PE was the main cause of death (similar to our study), while cancer, CHF, and respiratory disorders were the main causes of death in RPE patients [18]. Mortality of PE at 90 days greatly varies, from 1.5% [36] to 21.5% [15].

### Mortality during follow-up of patients with PE and RPE

In previous studies [13, 17, 34–37], as well as the present study, mortality in PE and RPE patients progressively increased during follow up. The most frequent causes of death were cancer, CHF, and COPD. The reason for this finding appears to be due to comorbidities (particularly cancer and vascular disease) that are present in RPE patients. While comparing confirmed PE and ruled out PE there was higher mortality in oncologic patients, followed by those in the unprovoked and provoked group.

### Complications during follow-up

VTE and anticoagulation are two of the main independent risk factors for recurrence of VTE and bleeding. In our cohort, new events of VTE and major bleeding were much higher in patients with confirmed PE than in RPE patients. This finding reflects “real clinical practice” because patients were not selected owing to the observational nature of the study, and a systematic search of asymptomatic VTE events was not performed. However, the high frequency of bleeding that was detected in RPE patients may be explained by the presence of multiple risk factors for bleeding (cancer and chemotherapy), which determined their initial inclusion in the suspected PE cohort.

We found no increase in the diagnosis of new cancer cases in patients with confirmed PE, either in the entire cohort, or in the unprovoked subgroup, which is consistent with previous reports [39, 40]. This association has been particularly described for selected patients with recurrent unprovoked PE, anemia, and bilateral DVT [39, 41, 42]. One explanation for our finding may be the real lack of an association between PE and new cancer. However, the number of unprovoked patients may have been insufficient to detect this association.

### Limitations of our study

Physicians from our hospital follow the European Society of Cardiology Guidelines in the diagnostic evaluation of suspected PE. However, because we performed an observational study, we were not able to report the chronological diagnostic test algorithm that was used by each physician. Our study was conducted in a single center with a high prevalence of cancer. Our cohort had a prevalence of cancer of 40%, while Maestre et al. [43] reported 25% and Bertoletti reported 7% [38]. Therefore, these results should only be extrapolated to centers with similar patients. Notably, the extent of neoplasm screening in suspected PE is still poorly standardized in the literature and depends on the treating physician.

### Strengths of our study

The main strength of this study was the design of a prospective cohort with a standardized follow-up of all suspected PE incident cases that were generated in real time. The cases were prospectively collected for the IRTD in a standardized data collection form and follow-up ensured high quality of data by minimizing the loss of events. As an observational study, our study reflected the current medical practice for patients with suspected PE at a teaching tertiary care hospital. Finally, to the best of our knowledge, no other studies have included such a large numbers of patients with suspected PE or such prolonged follow-up times.

## Conclusion

This study investigated the epidemiological and clinical characteristics, and short-term and long-term prognoses of patients with suspected PE. Overall mortality was similar between patients with confirmed PE and RPE patients, probably because of the more frequent or more severe comorbidities in RPE patients. These findings are relevant for understanding the prognosis of patients with suspected PE in everyday medical practice and could help physicians at the relevant decision-making time.

## References

[1] Geerts WH, Bergqvist D, Pineo GF, Heit JA, Samama CM, Lassen MR, Colwell CW (2008). Prevention of venous thromboembolism: American College of Chest Physicians Evidence-Based Clinical Practice Guidelines (8th Edition). Chest.

[2] Girard P, Sanchez O, Leroyer C, Musset D, Meyer G, Stern JB, Parent F (2005). Deep venous thrombosis in patients with acute pulmonary embolism: prevalence, risk factors, and clinical significance. Chest.

[3] Lobo JL, Zorrilla V, Aizpuru F, Uresandi F, Garcia-Bragado F, Conget F, Monreal M (2006). Clinical syndromes and clinical outcome in patients with pulmonary embolism: findings from the RIETE registry. Chest.

[4] Laporte S, Mismetti P, Decousus H, Uresandi F, Otero R, Lobo JL, Monreal M (2008). Clinical predictors for fatal pulmonary embolism in 15,520 patients with venous thromboembolism: findings from the Registro Informatizado de la Enfermedad TromboEmbolica venosa (RIETE) Registry. Circulation.

[5] Wells PS, Ginsberg JS, Anderson DR, Kearon C, Gent M, Turpie AG, Bormanis J, Weitz J, Chamberlain M, Bowie D, Barnes D, Hirsh J (1998). Use of a clinical model for safe management of patients with suspected pulmonary embolism. Ann Intern Med.

[6] Le Gal G, Righini M (2006). Pulmonary embolism in patients with unexplained exacerbations of chronic obstructive pulmonary disease. Ann Intern Med.

[7] Le Gal G, Righini M, Roy PM, Sanchez O, Aujesky D, Bounameaux H, Perrier A (2006). Prediction of pulmonary embolism in the emergency department: the revised Geneva score. Ann Intern Med.

[8] Baglin TP, White K, Charles A (1997). Fatal pulmonary embolism in hospitalised medical patients. J Clin Pathol.

[9] Righini M, Le Gal G, Aujesky D, Roy PM, Sanchez O, Verschuren F, Rutschmann O, Nonent M, Cornuz J, Thys F, Le Manach CP, Revel MP, Polletti PA, Meyer G, Mottier D, Perneger T, Bounameaux H, Perrier A (2008). Diagnosis of pulmonary embolism by multidetector CT alone or combined with venous ultrasonography of the leg: a randomised non-inferiority trial. Lancet.

[10] The PIOPED, Investigators (1990). Value of the ventilation/perfusion scan in acute pulmonary embolism. Results of the prospective investigation of pulmonary embolism diagnosis (PIOPED). JAMA.

[11] Worsley DF, Alavi A (1995). Comprehensive analysis of the results of the PIOPED Study. Prospective Investigation of Pulmonary Embolism Diagnosis Study. J Nucl Med.

[12] van Beek EJ, Tiel-van Buul MM, Buller HR, van Royen EA, ten Cate JW (1993). The value of lung scintigraphy in the diagnosis of pulmonary embolism. Eur J Nucl Med.

[13] Akram AR, Cowell GW, Logan LJ, Macdougall M, Reid JH, Murchison JT, Simpson AJ (2009). Clinically suspected acute pulmonary embolism: a comparison of presentation, radiological features and outcome in patients with and without PE. QJM.

[14] Forauer AR, McLean GK, Wallace LP (1998). Clinical follow-up of patients after a negative digital subtraction pulmonary arteriogram in the evaluation of pulmonary embolism. J Vasc Interv Radiol.

[15] van Strijen MJ, Bloem JL, de Monye W, Kieft GJ, Pattynama PM, van den Berg-Huijsmans A, Huisman MV, Antelope-Study G (2005). Helical computed tomography and alternative diagnosis in patients with excluded pulmonary embolism. J Thromb Haemost: JTH.

[16] Poulsen SH, Noer I, Moller JE, Knudsen TE, Frandsen JL (2001). Clinical outcome of patients with suspected pulmonary embolism. A follow-up study of 588 consecutive patients. J Intern Med.

[17] Carson JL, Kelley MA, Duff A, Weg JG, Fulkerson WJ, Palevsky HI, Schwartz JS, Thompson BT, Popovich J, Hobbins TE, Spera MA, Alavi A, Terrin ML (1992). The clinical course of pulmonary embolism. N Engl J Med.

[18] Roy PM, Meyer G, Vielle B, Le Gall C, Verschuren F, Carpentier F, Leveau P, Furber A (2006). Appropriateness of diagnostic management and outcomes of suspected pulmonary embolism. Ann Intern Med.

[19] Goldhaber SZ, Visani L, De Rosa M (1999). Acute pulmonary embolism: clinical outcomes in the International Cooperative Pulmonary Embolism Registry (ICOPER). Lancet.

[20] Kucher N, Rossi E, De Rosa M, Goldhaber SZ (2006). Massive pulmonary embolism. Circulation.

[21] Cohen AT, Agnelli G, Anderson FA, Arcelus JI, Bergqvist D, Brecht JG, Greer IA, Heit JA, Hutchinson JL, Kakkar AK, Mottier D, Oger E, Samama MM (2007). Venous thromboembolism (VTE) in Europe. The number of VTE events and associated morbidity and mortality. Thromb Haemost.

[22] Mazzei JA, Campos AL, Melero MJ (2005). Frequency and incidence of venous thromboembolism in a general hospital. Medicina (B Aires).

[23] White RH (2003). The epidemiology of venous thromboembolism. Circulation.

[24] Flinterman LE, van Hylckama VA, Cannegieter SC, Rosendaal FR (2012). Long-term survival in a large cohort of patients with venous thrombosis: incidence and predictors. PLoS Med.

[25] Vázquez F, Posadas-Martínez M, Vicens J, González Bernaldo de Quirós F, Giunta D (2013). Incidence rate of symptomatic venous thromboembolic disease in patients from a medical care program in Buenos Aires, Argentina: a prospective cohort. Thromb J.

[26] Wells PS, Anderson DR, Rodger M, Ginsberg JS, Kearon C, Gent M, Turpie AG, Bormanis J, Weitz J, Chamberlain M, Bowie D, Barnes D, Hirsh J (2000). Derivation of a simple clinical model to categorize patients probability of pulmonary embolism: increasing the models utility with the SimpliRED D-dimer. Thromb Haemost.

[27] Posadas-Martinez ML, Vazquez FJ, Giunta DH, Waisman GD, de Quiros FG, Gandara E (2014). Performance of the Wells score in patients with suspected pulmonary embolism during hospitalization: a delayed-type cross sectional study in a community hospital. Thromb Res.

[28] Boutitie F, Pinede L, Schulman S, Agnelli G, Raskob G, Julian J, Hirsh J, Kearon C (2011). Influence of preceding length of anticoagulant treatment and initial presentation of venous thromboembolism on risk of recurrence after stopping treatment: analysis of individual participants' data from seven trials. BMJ.

[29] Becattini C, Agnelli G, Schenone A, Eichinger S, Bucherini E, Silingardi M, Bianchi M, Moia M, Ageno W, Vandelli MR, Grandone E, Prandoni P, WARFASA investigators (2012). Aspirin for preventing the recurrence of venous thromboembolism. N Engl J Med.

[30] Verhovsek M, Douketis JD, Yi Q, Shrivastava S, Tait RC, Baglin T, Poli D, Lim W (2008). Systematic review: D-dimer to predict recurrent disease after stopping anticoagulant therapy for unprovoked venous thromboembolism. Ann Intern Med.

[31] Marcucci M, Iorio A, Douketis J (2013). Management of patients with unprovoked venous thromboembolism: an evidence-based and practical approach. Curr Treat Options Cardiovasc Med.

[32] Schulman S, Kearon C (2005). Definition of major bleeding in clinical investigations of antihemostatic medicinal products in non-surgical patients. J Thromb Haemost.

[33] Heit JA (2005). Venous thromboembolism: disease burden, outcomes and risk factors. J Thromb Haemost: JTH.

[34] Hull RD, Pineo GF, Brant RF, Mah AF, Burke N, Dear R, Wong T, Cook R, Solymoss S, Poon MC, Raskob G, Lite trial investigators (2006). Long-term low-molecular-weight heparin versus usual care in proximal-vein thrombosis patients with cancer. Am J Med.

[35] Heit JA, Silverstein MD, Mohr DN, Petterson TM, O'Fallon WM, Melton LJ (1999). Predictors of survival after deep vein thrombosis and pulmonary embolism: a population-based, cohort study. Arch Intern Med.

[36] Anderson FA, Wheeler HB, Goldberg RJ, Hosmer DW, Patwardhan NA, Jovanovic B, Forcier A, Dalen JE (1991). A population-based perspective of the hospital incidence and case-fatality rates of deep vein thrombosis and pulmonary embolism. The Worcester DVT Study. Arch Intern Med.

[37] van Beek EJ, Kuijer PM, Buller HR, Brandjes DP, Bossuyt PM, ten Cate JW (1997). The clinical course of patients with suspected pulmonary embolism. Arch Intern Med.

[38] Bertoletti L, Le Gal G, Aujesky D, Roy PM, Sanchez O, Verschuren F, Bounameaux H, Perrier A, Righini M (2011). Prognostic value of the Geneva prediction rule in patients in whom pulmonary embolism is ruled out. J Intern Med.

[39] Sorensen HT, Mellemkjaer L, Steffensen FH, Olsen JH, Nielsen GL (1998). The risk of a diagnosis of cancer after primary deep venous thrombosis or pulmonary embolism. N Engl J Med.

[40] Fennerty T (2001). Screening for cancer in venous thromboembolic disease. BMJ.

[41] Sorensen HT, Horvath-Puho E, Pedersen L, Baron JA, Prandoni P (2007). Venous thromboembolism and subsequent hospitalisation due to acute arterial cardiovascular events: a 20-year cohort study. Lancet.

[42] Prandoni P, Lensing AW, Buller HR, Cogo A, Prins MH, Cattelan AM, Cuppini S, Noventa F, ten Cate JW (1992). Deep-vein thrombosis and the incidence of subsequent symptomatic cancer. N Engl J Med.

[43] Maestre A, Trujillo-Santos J, Visona A, Lobo JL, Grau E, Maly R, Duce R, Monreal M, Investigators R (2014). D-dimer levels and 90-day outcome in patients with acute pulmonary embolism with or without cancer. Thromb Res.

[CR44] The pre-publication history for this paper can be accessed here: http://www.biomedcentral.com/1471-2466/14/200/prepub

